# 1-Phenyl-2-[4-(trifluoro­meth­yl)phen­yl]-1*H*-benzimidazole

**DOI:** 10.1107/S1600536813000834

**Published:** 2013-01-16

**Authors:** K. Jayamoorthy, T. Mohandas, P. Sakthivel, J. Jayabharathi

**Affiliations:** a Annamalai University, Chidambaram, Tamilnadu, India; b Shri Angalamman College of Engineering And Technology, Siruganoor, Tiruchirappalli, Tamilnadu 621 105, India; c Urumu Dhanalakshmi College, Tiruchirappalli, Tamilnadu 620 019, India

## Abstract

In the title mol­ecule, C_20_H_13_F_3_N_2_, the benzimidazole unit is close to being planar [maximum deviation = 0.012 (1) Å] and forms dihedral angles of 31.43 (7) and 61.45 (9)° with the 4-(trifluoromethyl)phenyl and 1-phenyl rings, respectively; the dihedral angle between these rings is 60.94 (10)°. In the crystal, C—H⋯F hydrogen bonds link the mol­ecules into chains along the *c*-axis direction. The CF_3_ group is rotationally disordered with an occupancy ratio of 0.557 (8):0.443 (8) for the F atoms.

## Related literature

For the properties of related compounds, see: Bu *et al.* (1996 ▶); Cross *et al.* (1995 ▶); Fu *et al.* (2011 ▶); Zhang *et al.* (2010 ▶). For bond lengths and angles in related structures, see: Yoon *et al.* (2011 ▶); Kassim *et al.* (2012 ▶).
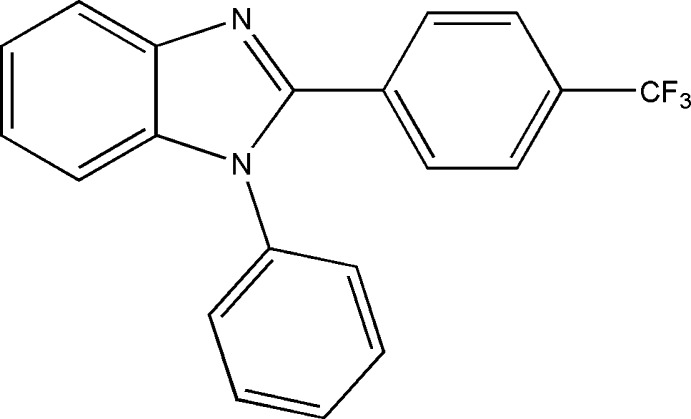


## Experimental

### 

#### Crystal data

C_20_H_13_F_3_N_2_
*M* *_r_* = 338.32
Triclinic, 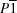

*a* = 8.7179 (4) Å
*b* = 9.6796 (5) Å
*c* = 11.3612 (6) Å
α = 67.654 (2)°
β = 68.123 (2)°
γ = 85.013 (2)°
*V* = 821.20 (7) Å^3^
*Z* = 2
Mo *K*α radiation
μ = 0.11 mm^−1^
*T* = 293 K
0.30 × 0.20 × 0.20 mm

#### Data collection

Bruker Kappa APEXII diffractometer
Absorption correction: multi-scan (*SADABS*; Bruker,2008 ▶) *T*_min_ = 0.960, *T*_max_ = 0.986
16592 measured reflections
2889 independent reflections
2338 reflections with *I* > 2σ(*I*)
*R*_int_ = 0.032

#### Refinement

*R*[*F* ^2^ > 2σ(*F* ^2^)] = 0.038
*wR*(*F* ^2^) = 0.105
*S* = 1.03
2889 reflections
255 parameters
36 restraints
H-atom parameters constrained
Δρ_max_ = 0.15 e Å^−3^
Δρ_min_ = −0.14 e Å^−3^

### 

Data collection: *APEX2* (Bruker, 2008 ▶); cell refinement: *APEX2* and *SAINT* (Bruker, 2008 ▶); data reduction: *SAINT*; program(s) used to solve structure: *SHELXS97* (Sheldrick, 2008 ▶); program(s) used to refine structure: *SHELXL97* (Sheldrick, 2008 ▶); molecular graphics: *PLATON* (Spek, 2009 ▶); software used to prepare material for publication: *PLATON*.

## Supplementary Material

Crystal structure: contains datablock(s) global, I. DOI: 10.1107/S1600536813000834/bv2218sup1.cif

Structure factors: contains datablock(s) I. DOI: 10.1107/S1600536813000834/bv2218Isup2.hkl

Supplementary material file. DOI: 10.1107/S1600536813000834/bv2218Isup3.cml

Additional supplementary materials:  crystallographic information; 3D view; checkCIF report

## Figures and Tables

**Table 1:** Hydrogen-bond geometry (Å, °)

*D*—H⋯*A*	*D*—H	H⋯*A*	*D*⋯*A*	*D*—H⋯*A*
C17—H17⋯F3^i^	0.93	2.54	3.429 (6)	160

Symmetry code: (i) 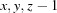.

**Fig. 1.**
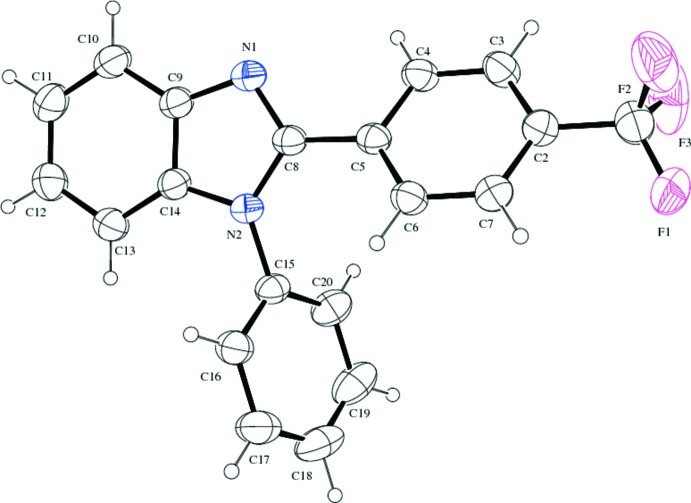
The molecular structure and labelling scheme for (I) with displacement ellipsoids for non-H atoms are drawn at the 30% probability level (major component only).

**Fig. 2.**
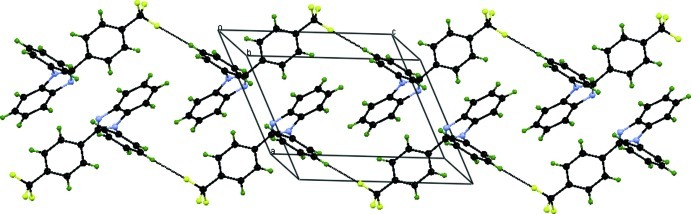
A packing diagram for (I) is drawn. Dashed lines indicate the intermolecular hydrogen bonding interactions.

## supplementary crystallographic information

### Comment

Recently much attention has been given by the researchers to crystals containing organic and inorganic ions due to their special structural features and their ferro electric properties (Fu *et al.*, 2011; Zhang *et al.*, 2010).

The imidazole ring can be easily accommodated with functional groups which allows the covalent incorporation of the NLO chromophores into polyimides leading to NLO side chain polymers (Bu *et al.*, 1996).

The NLO property of benzimidazole and its thermal stability in guest host systems have drawn our attention towards this chromophore (Cross *et al.*, 1995).

The asymmetric unit contains the title compound C_20_H_13_F_3_N_2_ in the space group P-1. The benzimidazole ring system in the molecule N1/N2/C8—C14 is essentially planar with maximum deviations of 0.012 (1) for N1. In the molecules the benzimidazole ring N1/N2/C8—C14 makes dihedral angles of 61.45 (9)° and 31.43 (7)° respectively with the phenyl ring C15—C20 and the trifluoromethyl substituted phenyl ring C2—C7.

The torsional angles of C1/C2/C3/C4 and C3/C4/C5/C8 are 178.14° and 179.82° respectively. The molecules are linked into chains by C—H···F hydrogen bond interactions along the c axis.

Bond lengths and bond angles are within normal range and are comparable to related structures (Yoon *et al.*, 2011; Kassim *et al.*, 2012)

### Experimental

To pure *N*-phenyl-*o*-phenylenediamine(17 mmol,3.128 g) in ethanol (10 ml) was added 4-(trifluoromethyl)-benzaldehyde (17 mmol, 2.38 ml) and ammonium acetate (3 g) were added while the temperature was maintained at 80°C. The reaction mixture was refluxed for 48 h and the reaction completion was monitored by TLC and finally it was extracted with dichloromethane. The separated solid was purified by column chromatography using petroleum ether as the eluent. Yield: 2.87 g (50%). Single crystals were grown in ethanol as solvent within a period of one week.

### Refinement

All the hydrogen atoms were geometrically fixed and allowed to ride on their parent atoms with C—H = 0.93 - 0.96 Å, and *U*_iso_ = 1.5_eq_(C) for methyl H atoms and 1.2*U*_eq_(C) for other H atoms.

The disordered trifluoromethyl was modelled with restrained bonds and angles based on the average values found for the non-disordered trifluoromethyl group with initial positions being derived from a difference map. In the final stages of refinement the group was refined as a riding and rotating group as for a methyl group.

### Crystal data

**Table:**

C_20_H_13_F_3_N_2_	*Z* = 2
*M**_r_* = 338.32	*F*(000) = 348
Triclinic, *P*1	*D*_x_ = 1.368 Mg m^−^^3^
Hall symbol: -P 1	Mo *K*α radiation, λ = 0.71073 Å
*a* = 8.7179 (4) Å	Cell parameters from 7057 reflections
*b* = 9.6796 (5) Å	θ = 2.3–27.1°
*c* = 11.3612 (6) Å	µ = 0.11 mm^−^^1^
α = 67.654 (2)°	*T* = 293 K
β = 68.123 (2)°	Block, colourless
γ = 85.013 (2)°	0.30 × 0.20 × 0.20 mm
*V* = 821.20 (7) Å^3^	

### Data collection

**Table:**

Bruker Kappa APEXII diffractometer	2889 independent reflections
Radiation source: fine focus sealed tube	2338 reflections with *I* > 2σ(*I*)
Graphite monochromator	*R*_int_ = 0.032
Detector resolution: 18.4 pixels mm^-1^	θ_max_ = 25.0°, θ_min_ = 2.3°
ω and φ scan	*h* = −10→10
Absorption correction: multi-scan (*SADABS*; Bruker,2008)	*k* = −11→11
*T*_min_ = 0.960, *T*_max_ = 0.986	*l* = −13→13
16592 measured reflections	

### Refinement

**Table:**

Refinement on *F*^2^	Primary atom site location: structure-invariant direct methods
Least-squares matrix: full	Secondary atom site location: difference Fourier map
*R*[*F*^2^ > 2σ(*F*^2^)] = 0.038	Hydrogen site location: inferred from neighbouring sites
*wR*(*F*^2^) = 0.105	H-atom parameters constrained
*S* = 1.03	*w* = 1/[σ^2^(*F*_o_^2^) + (0.0469*P*)^2^ + 0.1406*P*] where *P* = (*F*_o_^2^ + 2*F*_c_^2^)/3
2889 reflections	(Δ/σ)_max_ = 0.001
255 parameters	Δρ_max_ = 0.15 e Å^−^^3^
36 restraints	Δρ_min_ = −0.14 e Å^−^^3^

### Special details

**Table:**

Geometry. Bond distances, angles *etc*. have been calculated using the rounded fractional coordinates. All su's are estimated from the variances of the (full) variance-covariance matrix. The cell e.s.d.'s are taken into account in the estimation of distances, angles and torsion angles
Refinement. Refinement of *F*^2^ against ALL reflections. The weighted *R*-factor *wR* and goodness of fit *S* are based on *F*^2^, conventional *R*-factors *R* are based on *F*, with *F* set to zero for negative *F*^2^. The threshold expression of *F*^2^ > σ(*F*^2^) is used only for calculating *R*-factors(gt) *etc*. and is not relevant to the choice of reflections for refinement. *R*-factors based on *F*^2^ are statistically about twice as large as those based on *F*, and *R*- factors based on ALL data will be even larger.

### Fractional atomic coordinates and isotropic or equivalent isotropic displacement parameters (Å^2^)

**Table:**

	*x*	*y*	*z*	*U*_iso_*/*U*_eq_	Occ. (<1)
C1	−0.1427 (3)	0.2068 (3)	1.5614 (2)	0.0834 (5)	
C2	−0.04295 (19)	0.28680 (17)	1.41364 (16)	0.0618 (4)	
C3	0.0815 (2)	0.39161 (19)	1.37506 (17)	0.0672 (4)	
H3	0.1005	0.4140	1.4414	0.081*	
C4	0.17792 (19)	0.46346 (18)	1.23902 (17)	0.0614 (4)	
H4	0.2612	0.5346	1.2139	0.074*	
C5	0.15171 (17)	0.43049 (15)	1.13943 (15)	0.0523 (4)	
C6	0.02360 (19)	0.32742 (17)	1.17905 (16)	0.0590 (4)	
H6	0.0027	0.3061	1.1130	0.071*	
C7	−0.0730 (2)	0.25634 (18)	1.31517 (17)	0.0635 (4)	
H7	−0.1587	0.1876	1.3406	0.076*	
C8	0.25824 (17)	0.51116 (15)	0.99601 (15)	0.0524 (4)	
C9	0.40857 (19)	0.68454 (16)	0.81207 (16)	0.0575 (4)	
C10	0.4994 (2)	0.81574 (18)	0.71278 (18)	0.0711 (5)	
H10	0.5086	0.8970	0.7349	0.085*	
C11	0.5744 (2)	0.8215 (2)	0.58197 (19)	0.0782 (5)	
H11	0.6351	0.9086	0.5140	0.094*	
C12	0.5625 (2)	0.7008 (2)	0.54771 (18)	0.0774 (5)	
H12	0.6156	0.7090	0.4573	0.093*	
C13	0.4747 (2)	0.56999 (19)	0.64349 (17)	0.0682 (5)	
H13	0.4676	0.4887	0.6207	0.082*	
C14	0.39737 (18)	0.56482 (16)	0.77574 (15)	0.0549 (4)	
C15	0.27055 (17)	0.30532 (15)	0.90658 (15)	0.0539 (4)	
C16	0.1858 (2)	0.28353 (19)	0.83389 (17)	0.0663 (4)	
H16	0.1456	0.3645	0.7795	0.080*	
C17	0.1609 (2)	0.1411 (2)	0.8421 (2)	0.0855 (6)	
H17	0.1055	0.1259	0.7918	0.103*	
C18	0.2174 (3)	0.0223 (2)	0.9239 (3)	0.0940 (7)	
H18	0.1999	−0.0738	0.9296	0.113*	
C19	0.2999 (2)	0.0440 (2)	0.9977 (2)	0.0926 (7)	
H19	0.3362	−0.0377	1.0547	0.111*	
C20	0.3294 (2)	0.18642 (18)	0.9882 (2)	0.0743 (5)	
H20	0.3882	0.2016	1.0363	0.089*	
N1	0.32109 (16)	0.64829 (13)	0.95021 (13)	0.0604 (4)	
N2	0.29987 (15)	0.45366 (12)	0.89494 (12)	0.0530 (3)	
F1	−0.2223 (12)	0.0892 (8)	1.5887 (4)	0.142 (3)	0.557 (8)
F2	−0.2557 (8)	0.2984 (5)	1.6041 (5)	0.1329 (18)	0.557 (8)
F3	−0.0570 (6)	0.1792 (13)	1.6361 (4)	0.160 (3)	0.557 (8)
F1'	−0.2976 (8)	0.1695 (15)	1.5888 (7)	0.155 (4)	0.443 (8)
F2'	−0.1517 (17)	0.2719 (7)	1.6418 (4)	0.140 (3)	0.443 (8)
F3'	−0.0821 (14)	0.0771 (8)	1.6144 (5)	0.160 (3)	0.443 (8)

### Atomic displacement parameters (Å^2^)

**Table:**

	*U*^11^	*U*^22^	*U*^33^	*U*^12^	*U*^13^	*U*^23^
F1	0.203 (7)	0.116 (3)	0.0732 (19)	−0.092 (4)	−0.010 (3)	−0.018 (3)
F2	0.122 (3)	0.158 (4)	0.084 (2)	0.009 (3)	0.007 (2)	−0.053 (2)
F3	0.141 (3)	0.231 (7)	0.069 (2)	−0.037 (4)	−0.056 (2)	0.013 (4)
N1	0.0698 (8)	0.0499 (7)	0.0627 (8)	0.0024 (6)	−0.0207 (6)	−0.0258 (6)
N2	0.0609 (7)	0.0454 (7)	0.0545 (7)	0.0035 (5)	−0.0201 (6)	−0.0220 (6)
C1	0.0916 (14)	0.0895 (15)	0.0642 (12)	−0.0089 (12)	−0.0223 (11)	−0.0268 (11)
C2	0.0670 (10)	0.0589 (9)	0.0589 (10)	0.0050 (7)	−0.0227 (8)	−0.0224 (8)
C3	0.0728 (10)	0.0776 (11)	0.0603 (10)	0.0032 (8)	−0.0255 (8)	−0.0340 (9)
C4	0.0620 (9)	0.0639 (9)	0.0656 (10)	−0.0008 (7)	−0.0222 (8)	−0.0321 (8)
C5	0.0559 (8)	0.0482 (8)	0.0580 (9)	0.0100 (6)	−0.0232 (7)	−0.0247 (7)
C6	0.0647 (9)	0.0606 (9)	0.0600 (9)	0.0036 (7)	−0.0265 (8)	−0.0278 (8)
C7	0.0651 (9)	0.0593 (9)	0.0648 (10)	−0.0033 (7)	−0.0218 (8)	−0.0227 (8)
C8	0.0570 (8)	0.0473 (8)	0.0590 (9)	0.0084 (6)	−0.0240 (7)	−0.0248 (7)
C9	0.0622 (9)	0.0488 (8)	0.0607 (9)	0.0044 (7)	−0.0212 (7)	−0.0215 (7)
C10	0.0803 (11)	0.0526 (9)	0.0746 (12)	−0.0029 (8)	−0.0221 (9)	−0.0227 (8)
C11	0.0835 (12)	0.0625 (10)	0.0695 (12)	−0.0103 (9)	−0.0175 (9)	−0.0122 (9)
C12	0.0835 (12)	0.0795 (12)	0.0583 (10)	−0.0090 (10)	−0.0138 (9)	−0.0236 (9)
C13	0.0758 (10)	0.0674 (10)	0.0616 (10)	−0.0032 (8)	−0.0189 (8)	−0.0293 (9)
C14	0.0579 (8)	0.0492 (8)	0.0578 (9)	0.0039 (6)	−0.0209 (7)	−0.0206 (7)
C15	0.0528 (8)	0.0470 (8)	0.0604 (9)	0.0042 (6)	−0.0135 (7)	−0.0259 (7)
C16	0.0696 (10)	0.0668 (10)	0.0642 (10)	−0.0043 (8)	−0.0179 (8)	−0.0313 (8)
C17	0.0841 (12)	0.0859 (14)	0.0873 (14)	−0.0208 (11)	−0.0080 (10)	−0.0510 (12)
C18	0.0763 (12)	0.0638 (12)	0.1207 (18)	−0.0121 (10)	0.0082 (12)	−0.0525 (13)
C19	0.0771 (12)	0.0520 (10)	0.1213 (18)	0.0141 (9)	−0.0182 (12)	−0.0242 (11)
C20	0.0694 (10)	0.0558 (10)	0.0954 (13)	0.0136 (8)	−0.0318 (10)	−0.0268 (9)
F3'	0.229 (7)	0.098 (4)	0.084 (3)	0.048 (4)	−0.030 (4)	0.003 (2)
F2'	0.226 (8)	0.108 (4)	0.063 (2)	−0.050 (4)	−0.005 (4)	−0.042 (3)
F1'	0.107 (4)	0.209 (9)	0.089 (3)	−0.058 (4)	−0.023 (2)	0.008 (5)

### Geometric parameters (Å, º)

**Table:**

F1—C1	1.258 (9)	C11—C12	1.387 (3)
F1'—C1	1.319 (8)	C12—C13	1.369 (3)
F2—C1	1.346 (7)	C13—C14	1.382 (2)
F2'—C1	1.270 (8)	C15—C20	1.374 (2)
F3—C1	1.271 (6)	C15—C16	1.373 (2)
F3'—C1	1.328 (9)	C16—C17	1.377 (3)
N1—C8	1.312 (2)	C17—C18	1.363 (4)
N1—C9	1.380 (2)	C18—C19	1.369 (4)
N2—C8	1.3785 (19)	C19—C20	1.381 (3)
N2—C14	1.381 (2)	C3—H3	0.9300
N2—C15	1.428 (2)	C4—H4	0.9300
C1—C2	1.488 (3)	C6—H6	0.9300
C2—C3	1.377 (3)	C7—H7	0.9300
C2—C7	1.375 (2)	C10—H10	0.9300
C3—C4	1.377 (2)	C11—H11	0.9300
C4—C5	1.384 (2)	C12—H12	0.9300
C5—C8	1.469 (2)	C13—H13	0.9300
C5—C6	1.387 (2)	C16—H16	0.9300
C6—C7	1.377 (2)	C17—H17	0.9300
C9—C14	1.392 (2)	C18—H18	0.9300
C9—C10	1.390 (2)	C19—H19	0.9300
C10—C11	1.363 (3)	C20—H20	0.9300
			
C8—N1—C9	105.22 (13)	N2—C14—C13	131.82 (16)
C8—N2—C14	106.09 (12)	C9—C14—C13	122.50 (15)
C8—N2—C15	129.34 (12)	N2—C15—C16	119.38 (15)
C14—N2—C15	124.15 (12)	N2—C15—C20	119.91 (15)
F1—C1—F2	106.3 (5)	C16—C15—C20	120.70 (16)
F1—C1—F3	109.8 (6)	C15—C16—C17	119.60 (18)
F1—C1—C2	114.8 (3)	C16—C17—C18	120.1 (2)
F2—C1—F3	104.2 (6)	C17—C18—C19	120.2 (2)
F2—C1—C2	108.4 (3)	C18—C19—C20	120.4 (2)
F3—C1—C2	112.5 (3)	C15—C20—C19	118.9 (2)
F1'—C1—C2	114.9 (3)	C2—C3—H3	120.00
F2'—C1—C2	117.1 (4)	C4—C3—H3	120.00
F3'—C1—C2	112.0 (4)	C3—C4—H4	120.00
F1'—C1—F2'	104.8 (8)	C5—C4—H4	120.00
F1'—C1—F3'	103.7 (8)	C5—C6—H6	120.00
F2'—C1—F3'	102.8 (6)	C7—C6—H6	120.00
C1—C2—C3	119.76 (17)	C2—C7—H7	120.00
C1—C2—C7	120.63 (18)	C6—C7—H7	120.00
C3—C2—C7	119.62 (15)	C9—C10—H10	121.00
C2—C3—C4	120.44 (16)	C11—C10—H10	121.00
C3—C4—C5	120.38 (17)	C10—C11—H11	119.00
C4—C5—C6	118.73 (14)	C12—C11—H11	119.00
C4—C5—C8	117.86 (14)	C11—C12—H12	119.00
C6—C5—C8	123.37 (14)	C13—C12—H12	119.00
C5—C6—C7	120.67 (16)	C12—C13—H13	122.00
C2—C7—C6	120.13 (17)	C14—C13—H13	122.00
N1—C8—N2	112.81 (13)	C15—C16—H16	120.00
N1—C8—C5	122.94 (14)	C17—C16—H16	120.00
N2—C8—C5	124.24 (14)	C16—C17—H17	120.00
N1—C9—C10	130.14 (16)	C18—C17—H17	120.00
N1—C9—C14	110.21 (14)	C17—C18—H18	120.00
C10—C9—C14	119.65 (15)	C19—C18—H18	120.00
C9—C10—C11	117.88 (17)	C18—C19—H19	120.00
C10—C11—C12	121.69 (18)	C20—C19—H19	120.00
C11—C12—C13	121.68 (17)	C15—C20—H20	121.00
C12—C13—C14	116.59 (17)	C19—C20—H20	121.00
N2—C14—C9	105.68 (13)		
			
C9—N1—C8—C5	−178.47 (15)	C3—C4—C5—C8	179.82 (16)
C8—N1—C9—C10	179.79 (19)	C6—C5—C8—N1	147.01 (17)
C9—N1—C8—N2	0.13 (19)	C4—C5—C8—N2	150.81 (16)
C8—N1—C9—C14	−0.27 (19)	C8—C5—C6—C7	−179.41 (16)
C8—N2—C14—C9	−0.22 (18)	C4—C5—C6—C7	−1.7 (3)
C15—N2—C14—C9	−173.36 (15)	C4—C5—C8—N1	−30.8 (2)
C14—N2—C15—C20	114.16 (19)	C6—C5—C8—N2	−31.4 (3)
C14—N2—C8—N1	0.06 (19)	C5—C6—C7—C2	−0.2 (3)
C15—N2—C8—C5	−8.7 (3)	N1—C9—C10—C11	−179.91 (19)
C8—N2—C14—C13	179.45 (19)	C14—C9—C10—C11	0.2 (3)
C14—N2—C8—C5	178.63 (15)	N1—C9—C14—N2	0.31 (19)
C8—N2—C15—C20	−57.3 (2)	C10—C9—C14—N2	−179.74 (16)
C15—N2—C8—N1	172.71 (15)	C10—C9—C14—C13	0.6 (3)
C14—N2—C15—C16	−64.8 (2)	N1—C9—C14—C13	−179.40 (16)
C8—N2—C15—C16	123.79 (18)	C9—C10—C11—C12	−0.5 (3)
C15—N2—C14—C13	6.3 (3)	C10—C11—C12—C13	0.1 (3)
F1—C1—C2—C3	−162.7 (5)	C11—C12—C13—C14	0.6 (3)
F1—C1—C2—C7	16.9 (6)	C12—C13—C14—N2	179.49 (18)
F3—C1—C2—C3	−36.2 (7)	C12—C13—C14—C9	−0.9 (3)
F2—C1—C2—C3	78.6 (4)	N2—C15—C16—C17	178.39 (17)
F2—C1—C2—C7	−101.9 (4)	C20—C15—C16—C17	−0.5 (3)
F3—C1—C2—C7	143.4 (6)	N2—C15—C20—C19	−179.86 (19)
C7—C2—C3—C4	−1.4 (3)	C16—C15—C20—C19	−1.0 (3)
C3—C2—C7—C6	1.7 (3)	C15—C16—C17—C18	1.2 (3)
C1—C2—C3—C4	178.2 (2)	C16—C17—C18—C19	−0.3 (4)
C1—C2—C7—C6	−177.9 (2)	C17—C18—C19—C20	−1.2 (4)
C2—C3—C4—C5	−0.5 (3)	C18—C19—C20—C15	1.8 (4)
C3—C4—C5—C6	2.0 (3)		

### Hydrogen-bond geometry (Å, º)

**Table:**

*D*—H···*A*	*D*—H	H···*A*	*D*···*A*	*D*—H···*A*
C17—H17···F3^i^	0.93	2.54	3.429 (6)	160

Symmetry code: (i) *x*, *y*, *z*−1.
